# Case Report: Extending dosing intervals of biologics in adults severe asthma: a case series

**DOI:** 10.3389/falgy.2025.1635540

**Published:** 2026-01-21

**Authors:** Tomoya Harada, Miyu Nishigami, Hiroyuki Tanaka, Genki Inui, Hiroki Ishikawa, Hiroki Kohno, Yoshihiro Funaki, Tomohiro Sakamoto, Miki Takata, Ryota Okazaki, Masato Morita, Shin Kitatani, Akira Yamasaki

**Affiliations:** 1 Division of Respiratory Medicine and Rheumatology, Faculty of Medicine, Tottori University, Yonago, Tottori, Japan; 2 Nawa Clinic, Daisen-cho, Saihaku, Tottori, Japan; 3 Department of Respirology, Tottori Prefectural Kousei Hospital, Kurayoshi, Tottori, Japan

**Keywords:** asthma, biological drugs, cost, dose spacing, extended interval dosing

## Abstract

**Background:**

Biological therapies have improved the control of severe asthma. However, biological therapies are expensive. Therefore, the cost-effective use of these medications after achieving improved disease control warrants consideration.

**Methods:**

This retrospective case series analyzed 69 adult patients with asthma who received biological therapies at our department between 2009 and 2023. Among them, 11 patients underwent dosing interval extension. We collected data on their clinical characteristics, asthma control status, and changes in clinical parameters after interval extension.

**Results:**

At the time of dosing interval extension, the mean patient age was 62.1 years, with 5 female patients. Omalizumab was used in five cases, benralizumab in five, and dupilumab in one. The dosing intervals were extended by 1.5 to 2 times. The median duration from the initiation of biologics to interval extension was 7.0 months, with 6 patients undergoing extension within the first 7 months. One year after extension, asthma exacerbation occurred in only one patient receiving omalizumab. The frequency of exacerbations and the proportion of patients receiving oral corticosteroids (OCS) were decreased. The median ACT score remained at 25, while the median daily OCS dose decreased from 5.0 mg to 4.0 mg (prednisolone equivalent). The median % predicted FEV₁ changed from 84.9% to 78.3%.

**Conclusions:**

For patients whose asthma control improves after initiating biological therapy, extending the dosing interval may be a feasible treatment option. Given the retrospective, single-center design and small sample size, these findings are exploratory and generate hypotheses that require validation in larger, prospective studies.

## Introduction

Asthma is a common chronic respiratory disease characterized by various symptoms, such as wheezing, shortness of breath, chest tightness, cough, and expiratory airflow limitation (1). Asthma affects over 300 million people worldwide and is associated with significant morbidity and mortality (2). Most patients with asthma achieve good control with medications, such as inhaled corticosteroids (ICS), bronchodilators, and anti-inflammatory agents. However, approximately 3%–10% do not achieve adequate disease control despite medications (3–7). These patients are categorized as having severe asthma. Severe asthma is defined as asthma that is uncontrolled despite adherence to an optimized high-dose combination of ICS and long-acting beta-2 agonist (LABA) therapy, and management of contributory factors, or worsens when high-dose treatment is decreased. Recent advances in our understanding of the pathogenesis of asthma have revealed that it is primarily a chronic airway inflammation involving a type 2 immune response (8). Over the last few decades, several biologics targeting type 2 inflammation, such as immunoglobulin E (IgE), interleukin-4 (IL-4), interleukin-5 (IL-5) and thymic stromal lymphopoietin (TSLP), have been introduced for the treatment of severe asthma (9). The use of these biological agents is a significant paradigm shift in asthma disease control in medicine (10).

Biological therapies are currently approved for use in patients with severe asthma that remains uncontrolled despite medium-to-high doses of ICS/LABA and additional controller medications. These treatments have been shown to improve asthma control, lung function, and asthma-related QOL, as well as reduce exacerbations and the required dose of oral corticosteroids (OCS) (9, 10). Although biologics play a significant role in the management of severe asthma, they are expensive and the medical cost burden is high. After disease control improves, it is necessary to consider drug usage methods that support responsible healthcare economics.

The GINA (Global Initiative for Asthma) guidelines for the treatment of severe asthma state (11) that when there is a good response to treatment, the first step is to reduce or discontinue OCS, and then, consider reducing the dose of inhalers and other therapeutic agents. However, dose reduction of biologic agents has not been mentioned. Two recent studies have reported on the cessation of biologics: (1) the xolair persistency of response after long-term therapy (XPORT) study and (2) the continuing long-term mepolizumab treatment (COMET) study. The XPORT study was a randomized, double-blind, placebo-controlled withdrawal study that included subjects with moderate-to-severe persistent asthma receiving long-term omalizumab (12), while the COMET study was a randomized, double-blind, placebo-controlled, parallel-group, multicenter study that included with severe eosinophilic asthma receiving long-term mepolizumab (13). These studies reported that biologic cessation increased exacerbation and reduced asthma control. From a healthcare economics perspective, other strategies to reduce the use of biologic therapies include dose reduction and extension of dosing intervals. In Japan, with the exception of omalizumab, biologic therapies for asthma are available in a single dosage form, making dose reduction difficult. Therefore, extending the dosing interval is a practical approach for reducing treatment burden and costs in cases where the asthma is well-controlled. This study aimed to describe real-world outcomes after extending biologic dosing intervals in adults with well-controlled severe asthma.

## Patients and methods

We retrospectively reviewed the medical records of 69 patients who were prescribed biologics for the treatment of severe asthma at our hospital during the 14-year period from January 2009 to March 2023. Biologics were administered to patients with severe asthma whose asthma symptoms remained uncontrolled, despite the use of medium to high-dose ICS and multiple long-term management drugs. The aim was to improve asthma symptom control and enable dose reduction or discontinuation of OCS. Inclusion criteria comprised patients whose biologic therapy dosing intervals were extended for reasons other than adverse events, such as high medical costs. Patients who had not yet reached 12 months since the extension of biologic dosing intervals were excluded. This exclusion was applied to account for the seasonal variability of asthma, as exacerbation frequency may fluctuate throughout the year. A 12-month observation period was therefore deemed necessary to adequately evaluate the outcomes of dosing interval extension. In this study, dosing intervals were extended in 11 patients, all of whom completed a 12-month follow-up period without requiring a return to the original dosing regimen. The extension of the dosing interval for biologics was defined as administering the biologic medication at a dosing interval longer than the one prescribed for each product based on health insurance system. For each biologic, the dosing interval is as follows: for omalizumab, the dosing interval is determined based on body weight and IgE levels, with doses administered every 2 to 4 weeks; for benralizumab, 30 mg is administered every 8 weeks; and for dupilumab, 300 mg is administered every 2 weeks. The extension of the dosing interval for biologics was determined through discussions between the patient and physician, based on the patient's preferences. Notably, no specific conditions, such as favorable results in pulmonary function tests or other specific evaluations, were required when extending the dosing interval. The dosing interval regimen was not determined based on pharmacokinetic considerations such as half-life, but rather was decided through a shared decision-making process between the treating physician and the patient. This process referenced extended dosing interval regimens for biologic agents used in treating other diseases. Asthma exacerbation was defined as cases requiring systemic glucocorticoids, unscheduled visits, emergency room visits, and hospitalization due to asthma attacks. We evaluated the changes in asthma control after the prolonged use of biologics at extended dosing intervals and the retention rate of the dose interval regimen. We also evaluated the asthma-related treatment changes and examination findings, and respiratory function tests performed before and after a prolonged period of being treated with biologics at extended dosing intervals.

As the study group was small (*n* = 11), only descriptive statistics were used. No inferential statistical tests were performed. Continuous variables are presented as median [interquartile range (IQR)], and categorical variables as numbers and percentages.

This observational study was conducted according to the ethical standards of the Declaration of Helsinki and approved by the ethical review board of Tottori University Hospital (Tottori, Japan; registration number 21A060). Written informed consent was obtained from the individuals or their legal guardian for the publication of any potentially identifiable images or data included in this article.

## Results

The clinical characteristics and asthma treatment details of the 11 patients are summarized in Table 1. The median age at the time of dosing interval extension was 65 years [IQR, 47–80]; five patients (45.5%) were female, and seven (63.6%) were never smokers. Allergic rhinitis was present in six patients (54.5%), and chronic rhinosinusitis in eight (72.7%). All 11 patients received ICS/LABA combination therapy, and high-dose ICS were used in eight patients (72.7%), while five patients (45.5%) were receiving OCS, with a median daily dose of 5.0 mg [4.5–5.0] of prednisolone (PSL) equivalents. Details of the extended dosing regimens for biological therapies are presented in Table 2. The median duration from the initiation of biologics to the extension of dosing intervals was 7.0 months [6–39]. Five patients received omalizumab, five received benralizumab, and one received dupilumab. The reasons for extending the dosing interval included socioeconomic considerations (*n* = 7), stable asthma conditions (*n* = 2), and patient concerns about the side effects without actual adverse events (*n* = 2). Regarding socioeconomic considerations, biological therapies are extremely costly. Therefore, the extensions were implemented in response to patients' requests to reduce their financial burden.

**Table 1:** Patient background at the initiation of biologics in the extended dosing interval group.

Patient	Age	Sex	Body mass index	Smoking history	Comorbidities	Perennial antigen	High dose ICS	ICS/LABA	LAMA	LTRA	SRT	OCS	PSL dose (mg/day)
1	73	F	24.4	Never	AR, CRS, N-ERD	No	Yes	Yes	No	No	Yes	Yes	5.0
2	65	F	25.7	Past	AR, CRS, N-ERD	N/D	Yes	Yes	No	Yes	Yes	Yes	4.0
3	59	M	24.7	Past	AR, CRS	No	Yes	Yes	Yes	No	Yes	Yes	5.0
4	81	M	26.3	Never	CRS	No	Yes	Yes	No	Yes	Yes	No	
5	45	F	24.1	Past	None	Yes	Yes	Yes	Yes	Yes	No	Yes	5.0
6	73	F	23.8	Never	AR, CRS	Yes	Yes	Yes	No	Yes	No	No	
7	16	M	21.3	Never	AR	Yes	No	Yes	No	Yes	Yes	No	
8	83	M	21.7	Never	ECRS	No	Yes	Yes	No	Yes	No	No	
9	80	F	20.6	Never	CRS	No	No	Yes	No	Yes	No	Yes	5.0
10	61	M	22.2	Past	AR, COPD	Yes	No	Yes	No	Yes	Yes	No	
11	47	M	24.1	Never	ECRS	No	Yes	Yes	Yes	Yes	Yes	No	

AR, allergic rhinitis; CRS, chronic sinusitis; ECRS, eosinophilic chronic sinusitis; N-ERD, NSAIDs-exacerbated respiratory disease; COPD, chronic obstructive pulmonary disease; ICS, inhaled corticosteroid; LABA, long-acting β2 agonist; LAMA, long-acting muscarinic antagonist; LTRA, Leukotriene receptor antagonist; SRT, Sustained release theophylline; PSL, prednisolone.

**Table 2:** Types of biologics dosage, administration interval, and extended regimen.

Patient	Biologics	Original dose and interval	Duration until extended	Extended dose interval regimen
1	Omalizumab	450 mg, Q4W	39 months	300 mg, Q6W
2	Omalizumab	300 mg, Q4W	41 months	300 mg, Q8W
3	Benralizumab	30 mg, Q8W	6 months	30 mg, Q12W
4	Benralizumab	30 mg, Q8W	17 months	30 mg, Q12W
5	Dupilumab	300 mg, Q2W	0 month	300 mg, Q4W
6	Omalizumab	300 mg, Q2W	39 months	300 mg, Q4W
7	Omalizumab	300 mg, Q2W	3 months	300 mg, Q4W
8	Benralizumab	30 mg, Q8W	7 months	30 mg, Q12W
9	Benralizumab	30 mg, Q8W	7 months	30 mg, Q12W
10	Omalizumab	525 mg, Q4W	41 months	525 mg, Q8W
11	Benralizumab	30 mg, Q8W	7 months	30 mg, Q16W

Q4W, every 4 weeks; Q6W, every 6 weeks; Q8W, every 8 weeks; Q12W, every 12 weeks; Q16W, every 16 weeks.

**Figure 1:**
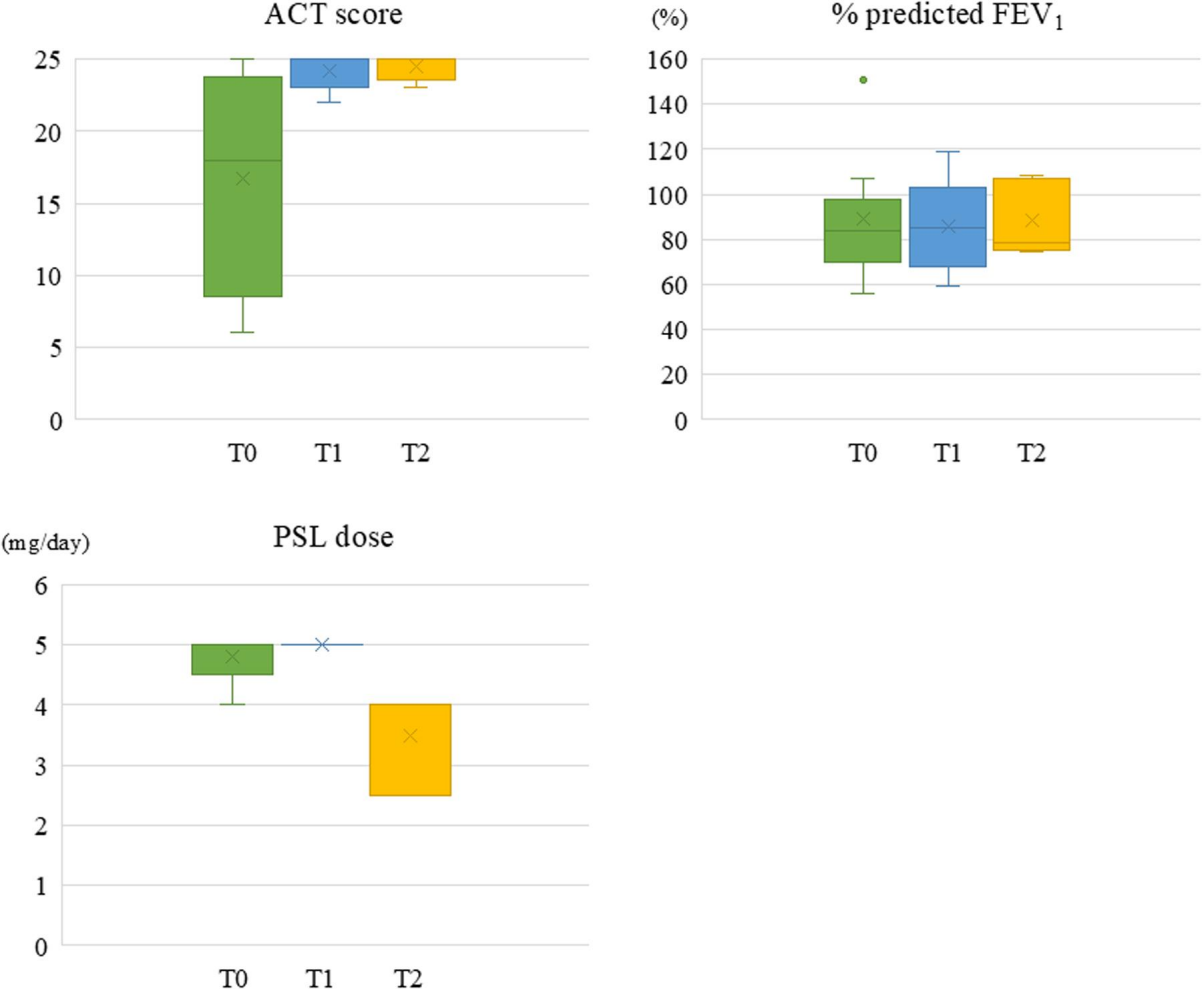
Temporal changes in ACT score, % predicted FEV_1,_ and daily prednisolone dose. This figure shows the median values and ranges of the ACT score, % predicted FEV_1,_ and daily prednisolone dose at three time points. T0: Before starting of biologics, T1: At the start of extended biologics, T2: 1 year after extended biologics. ACT, asthma control test; FEV_1_, forced expiratory volume in 1 s; PSL, prednisolone.

Detailed data on the Asthma Control Test (ACT) scores, blood eosinophil counts, serum IgE levels, pulmonary function test, presence and frequency of asthma exacerbations, and the doses of ICS and OCS at three time points—before initiation of biologics, at the time of dosing interval extension, and one year after the extension—are presented in Supplementary Table S1. Summary of clinical parameters are shown in Table 3. The median ACT score was 25 [23–25] at the time of dosing interval extension and remained stable at 25 [23.5–25] after one year. The median blood eosinophil count decreased from 244 cells/μL [94–864] before biologic initiation to 134 cells/μL [0–390] at the time of interval extension, and was 222 cells/μL [0–620] after one year. The median percentage of predicted forced expiratory volume 1 (FEV_1_) slight decrease from 84.9% [67.1–103.1] at the time of extension to 78.3% [75.2–106.9] after one year, while the FEV_1_/ forced vital capacity (FVC) ratio showed a slight increase from 62.3% [55.0–74.2] to 63.6% [55.7–70.4]. Regarding asthma exacerbations within one year, 10 patients (90.9%) had experienced exacerbations before biologic initiation, compared to 3 patients (27.3%) at the time of dosing interval extension and only 1 patient (9.1%) one year later. The annual number of exacerbations decreased from a median of 3.0 [2.0–6.0] before biologics to 0.0 [0.0–0.0] at the time of extension, and further to 0.0 [0.0–0.0] after one year. In terms of pharmacologic therapy, the number of patients receiving OCS decreased from 5 (45.5%) before biologics to 3 (27.3%) both at the time of extension and one year later. The median daily PSL dose also decreased from 5.0 mg [5.0–5.0] at the time of extension to 4.0 mg [2.5–4.0] after one year. Similarly, the number of patients receiving high-dose ICS declined from 8 (72.7%) before biologics to 7 (63.6%) at the time of extension and 6 (54.5%) after one year. Figure 1 shows the temporal changes in ACT score, % predicted FEV₁, and daily prednisolone dose.

**Table 3:** Summary of clinical parameters before biologics administration, at the time of dosing interval extension, and one year after the extension of the dosing interval.

Parameter	Before biologics administration (T0)	At interval extension (T1)	1 year after extension (T2)	ΔT1−T0	ΔT2−T1
Annual number of asthma exacerbation	3.0 (2.0, 6.0)	0 (0, 0)	0 (0, 0)	-3 (-6, -1)	0 (0, 0)
ACT score	18 (9, 24)	25 (23, 25)	25 (23.5, 25)	4 (0, 4)	0.5 (0, 1)
BEC (/μL)	244 (94, 864)	134 (0, 390)	222 (0, 620)	-244 (-741, 350)	0 (-95, 0)
IgE (IU/mL)	277 (233, 584)	186 (117, 714)	556 (32, 556)	-45 (-89, 0)	-2 (-8, -2)
FEV_1_ (L)	1.90 (1.49, 2.81)	1.87 (1.29, 2.91)	2.32 (1.50, 2.98)	0.03 (-0.09, 0.28)	0.03 (-0.35, 0.26)
%FEV_1_ (%)	83.7 (69.7, 97.9)	84.9 (67.1, 103.1)	78.3 (75.2, 106.9)	1.7 (-4.8, 10.4)	2.3 (-11.2, 8.1)
FEV_1_/FVC (%)	66.9 (60.1, 69.8)	62.3 (55.0, 74.2)	63.6 (55.7, 70.4)	0.1 (-5.2, 2.2)	2.4 (-5.0, 5.0)
PSL dose (mg/day)	5.0 (4.5, 5.0)	5.0 (5.0, 5.0)	4.0 (2.5, 4.0)	0 (0, 0)	0 (-1, 0)

Values are presented as medians (interquartile ranges).

ACT, asthma control test; BEC, blood eosinophil counts; FEV_1_, forced expiratory volume in 1 s; FVC, forced vital capacity; PSL, prednisolone.

These findings indicate that extending the dosing interval of biologics did not lead to worsening of asthma control; rather, it was associated with a continued reduction in exacerbation frequency and allowed for tapering of concomitant asthma medications.

## Discussion

This study describes the clinical characteristics of 11 severe asthma patients who underwent extended dosing intervals of biologics, as well as the findings after the dosing interval extension, asthma exacerbations, and changes in asthma treatment. Only one patient experienced an asthma exacerbation after the dosing interval extension, and the dosages of both OCS and ICS were reduced.

The efficacy of biologics is well-established, and clinical data in real-world settings are accumulating. Clinically, the REALITI-A study (14), REDES study (15), ORBEII study (16) and CROSSROAD study (17) showed that mepolizumab, benralizumab, and dupilumab were all effective for treating severe asthma. Additionally, the effectiveness of biologics for asthma treatment has been verified and demonstrated in various real-world settings (18, 19). However, biologics are expensive, and their long-term use may lead to health economics issues. Recently, cost-effectiveness has become an important consideration as biologics are costly despite their effectiveness. A recent review on the cost-effectiveness of biologics for allergic diseases concluded that, although biologics have transformed the management of severe allergic conditions, ongoing evaluation of their economic impact and sustainability is crucial (20). Therefore, the subsequent use of biologics in patients with improved asthma control is very important. The GINA guidelines recommend reducing or discontinuing OCS initially, and then, step-down treatment when asthma control improves (11). However, the guidelines did not mention biologics.

Clinical trials aimed at treatment discontinuation after improved asthma control have been conducted using omalizumab (*n* = 1) and mepolizumab (*n* = 2) (12, 13, 21). In all cases, the drugs were administered for more than one year before discontinuation. The results showed worsening asthma control and an increase in asthma exacerbation frequency among patients, indicating that the discontinuation of biologics is challenging, even after long-term administration. Discontinuation of biologics has also been investigated in patients with rheumatoid arthritis (22, 23). Evidence from these studies suggests that when biologics are introduced early after disease onset and deep remission is achieved while on medications, remission can be maintained even after discontinuation (22, 23). In contrast, many trials have shown that control worsens after the discontinuation of biologics, while other studies have shown that remission can be maintained with dose reduction (24–27). Consequently, current guidelines recommend dose reduction or extending the dosing interval of biologics, rather than discontinuation, once remission is achieved (28). Clinically, the use of dupilumab at extended dosing intervals for atopic dermatitis has been investigated. Various studies including retrospective trials, randomized controlled trials, and patient-centered trials have shown that treatment efficacy can be maintained even after extending the dosing interval (29, 30). Conversely, some studies have shown a reduction in treatment efficacy with an extension of the dosing interval (31). For example, the subcutaneous administration (300 mg every 4 weeks) of mepolizumab has been approved for treating eosinophilic granulomatosis with polyangiitis. Prospective and retrospective studies, as well as real-life setting studies, which examined the efficacy of a reduced dose (100 mg every 4 weeks) (32, 33) have all reported a remission rate of 80%–90%.

In the field of asthma, the extension of dosing intervals for biologic has also been investigated. One study compared the extension and dose reduction of omalizumab (34), one study examined the extension of dosing intervals for mepolizumab (35), and one study investigated the extension of dosing intervals for anti-IL-5 treatments (36). The study on omalizumab was a retrospective analysis, where all patients in the dose reduction group experienced exacerbations, and 27% in the dosing interval extension group experienced exacerbations, with a median time to exacerbation of 8 months. The study on mepolizumab was also a retrospective analysis, and no deterioration in ACT or lung function, nor any asthma exacerbations, were observed within one year after the extension of dosing intervals. The study on anti-IL-5 treatment was an RCT, and the exacerbation rate at 52 weeks showed that 32% of patients in the dosing interval extension group and 17% in the standard dosing group experienced exacerbations. While there was no significant difference, the dosing interval extension group tended to have more exacerbations. In this RCT, the protocol permitted a 50% extension to the dosing interval provided that asthma control was maintained. If this stability persisted, the interval could be extended by a further 50%. In cases of exacerbation, however, the interval was shortened. The extended dosing intervals used in this study were comparable to those used in our study and in previous reports. All studies included cases that did not require OCS for exacerbations. Based on the RCT and the omalizumab study, the extension of dosing intervals may not be useful, as exacerbations occurred even with extended intervals. However, the study on mepolizumab showed no exacerbations. In our study, only one exacerbation requiring OCS occurred over the course of one year, indicating a good result.

One of the distinguishing features of our study compared with previous studies is that it included a case treated with dupilumab. Furthermore, unlike other studies, we did not apply predefined criteria for selecting patients eligible for dosing interval extension. In prior studies, eligibility often required stable asthma control for at least one year before extending dosing intervals. In contrast, in our study, dosing interval extension was determined through shared decision-making between the physician and the patient, often influenced by factors such as medical costs, thereby reflecting a more real-world clinical practice. Another noteworthy aspect of our study is the variability in the timing of dosing interval extension. While previous studies typically extended intervals after at least one year of biologic therapy, in our cohort, six patients (54.5%) underwent interval extension within seven months of biologic initiation. These findings suggest that long-term administration of biologics may not be a prerequisite for maintaining asthma control after implementing extended dosing intervals.

Studies on the discontinuation of biologics have reported that asthma control deteriorates early when treatment is discontinued after achieving good asthma control through the long-term administration of mepolizumab (13, 21). Therefore, factors other than the dosing interval may influence the success or failure of extending the dosing interval. In this study, at the time of extending the dosing interval, the median ACT score was 25, indicating very good asthma control. Likewise, lung function was preserved, with a median % predicted FEV_1_ of 84.9%, which falls within the normal range. For rheumatoid arthritis, extending the dosing interval or reducing the dosage of biologics is recommended after achieving remission (28). Similarly, for asthma, achieving good disease control including lung function (37–39), and introducing biological therapy at an earlier stage may be a necessary factor for the successful extension of dosing intervals. Regarding the extent of dosing interval extension, based on this study, an extension of up to twice the original interval was achievable, and this may serve as a useful reference point.

This study has several limitations. First, it was a retrospective study conducted at a single center with a small sample size, and complete longitudinal data were not available for all patients. In addition, as a case series, the study lacked a control group, which limits the ability to draw comparative conclusions and precludes direct efficacy comparisons with patients who maintain standard dosing intervals. Thus, the observed outcomes may reflect patient selection bias because only patients for whom biologics were highly effective and whose physicians deemed interval extension feasible were included. Furthermore, since this cohort primarily consisted of low-risk, highly adherent patients, it may not be representative of the general severe asthma population. Second, the dosing interval was extended based on the judgment of the attending physician in consultation with the patient, and thus, the extended dosing intervals were not consistent between patients. Therefore, only patients for whom biologics were highly effective, and whose attending physicians determined that extending the dosing interval was feasible, were included in this study. Additionally, the most appropriate dosing interval extension was not determined. Third, since this was a retrospective study, some cases had missing data regarding ACT scores, pulmonary function tests, and serum IgE levels. This missing data may have affected the interpretation of the results. Given these methodological limitations, the present findings should be considered as hypothesis-generating and require prospective validation in larger, multicenter studies.

In conclusion, extending the dosing interval of biologics may represent a viable treatment option associated with favorable clinical outcomes and potential economic benefits for patients with asthma. Notably, even a relatively short duration of biologic therapy may be sufficient for interval extension if asthma control, including pulmonary function, is well maintained. To validate these findings, large-scale prospective studies are warranted.

## Data Availability

The raw data supporting the conclusions of this article will be made available by the authors, without undue reservation.
