# Supplementary material for: Case Report: Extending dosing intervals of biologics in adults severe asthma: a case series
Source: Front Allergy. 2026 Jan 21;6:1635540. doi: 10.3389/falgy.2025.1635540 (PMC12868160; doi:10.3389/falgy.2025.1635540)
Supplement: Supplementary file 1 [file Table1.docx]

**TABLE 3** Biomarkers, pulmonary function tests, corticosteroid dose, and exacerbation before biologics administration, at the time of dosing interval extension, and one year after the extension of the dosing interval

| Patient | Time point | Asthma exacerbation | times | ACT | BEC  (/μL) | IgE  (IU/mL) | FEV_1_  (L) | %FEV_1_  (%) | FEV_1_/FVC  (%) | ICS dose | PSL dose  (mg/day) |
| --- | --- | --- | --- | --- | --- | --- | --- | --- | --- | --- | --- |
| 1 | T0  T1  T2 | Yes  No  No | 2  0  0 | N/D  N/D  N/D | 0  350  148 | 519  N/D  556 | 1.37  1.30  N/D | 81.1  82.3  N/D | 65.6  62.8  N/D | High  High  High | 5.0  0  0 |
| 2 | T0  T1  T2 | Yes  Yes  No | 2  4  0 | N/D  N/D  N/D | 0  520  630 | 277  N/D  386 | 2.02  1.24  N/D | 96.7  62.6  N/D | 60.1  55.1  N/D | High  High  High | 4.0  0  0 |
| 3 | T0  T1  T2 | Yes  Yes  No | 18  2  0 | 6  N/D  N/D | 244  0  N/D | 193  112  N/D | 1.67  1.76  N/D | 56.0  59.1  N/D | 54.8  56.8  N/D | High  High  High | 5.0  5.0  4.0 |
| 4 | T0  T1  T2 | Yes  No  No | 6  0  0 | N/D  25  25 | 2222  0  N/D | 233  133  N/D | 1.49  1.98  N/D | 64.5  88.8  N/D | 72.0  79.2  N/D | High  High  High | 0  0  0 |
| 5 | T0  T1  T2 | Yes  Yes  No | 3  3  0 | 25  25  N/D | 148  148  N/D | 2699  2699  N/D | 1.70  1.70  1.80 | 69.7  69.7  75.6 | 52.0  52.0  61.9 | High  High  High | 5.0  5.0  4.0 |
| 6 | T0  T1  T2 | Yes  No  Yes | 6  0  1 | N/D  N/D  N/D | 94  1131  1185 | 584  N/D  N/D | 1.39  1.23  1.19 | 83.7  79.4  78.3 | 69.8  61.8  63.6 | High  Medium  Medium | 0  0  0 |
| **7** | T0  T1  T2 | Yes  No  No | N/D  0  0 | N/D  N/D  N/D | 134  134  N/D | 424  424  N/D | 3.30  3.35  3.56 | 97.9  100.0  105.6 | 69.6  69.8  73.1 | Medium  Medium  Medium | 0  0  0 |
| 8 | T0  T1  T2 | Yes No  No | 2  0  0 | 20  24  25 | 741  0  0 | 245  224  N/D | 1.90  2.72  2.40 | 83.0  118.8  108.1 | 69.6  72.5  67.6 | High  High  Medium | 0  0  0 |
| 9 | T0  T1  T2 | No  No  No | 0  0  0 | N/D  22  23 | 390  0  0 | 108  40  32 | 2.06  N/D  N/D | 150.4  N/D  N/D | 66.9  N/D  N/D | Medium  Medium  Medium | 5.0  5.0  2.5 |
| 10 | T0  T1  T2 | Yes  No  No | 4  0  0 | N/D  N/D  N/D | 864  390  295 | 672  811  1323 | 2.81  2.76  2.32 | 93.7  87.6  74.8 | 60.3  54.7  49.4 | Medium  Medium  Medium | 0  0  0 |
| 11 | T0  T1  T2 | Yes  No  No | 3  0  0 | 16  25  25 | 1224  0  N/D | 238  147  N/D | 3.96  4.17  N/D | 106.7  112.4  N/D | 79.8  81.0  N/D | High  High  High | 0  0  0 |

OCS dose is expressed as prednisolone equivalent (mg/day).

T0: Before starting of biologics, T1: At the start of extended biologics, T2: 1 year after extended biologics

N/D: Not done, ACT: Asthma control test, BEC: blood eosinophil counts, FEV_1_: forced expiratory volume in 1 second, FVC: forced vital capacity, ICS: inhaled corticosteroid, PSL: prednisolone
